# Epidemiological and clinical aspects of sporotrichosis in patients seen at a reference hospital in Madagascar

**DOI:** 10.1371/journal.pntd.0011478

**Published:** 2023-07-26

**Authors:** Fandresena Arilala Sendrasoa, Volatantely Tobiniaina Ratovonjanahary, Irina Mamisoa Ranaivo, Tahinamandranto Rasamoelina, Mendrika Fifaliana Rakotoarisaona, Malalaniaina Andrianarison, Lala Soavina Ramarozatovo, Fahafahantsoa Rapelanoro Rabenja

**Affiliations:** 1 Department of dermatology, Faculty of Medicine, University of Antananarivo, Antananarivo, Madagascar; 2 Centre d’Infectiologie Charles Mérieux, University of Antananarivo, Antananarivo, Madagascar; Universidade Federal do Para, BRAZIL

## Abstract

**Introduction:**

Sporotrichosis is a subacute to chronic fungal infection of skin and subcutaneous tissues, caused by dimorphic fungi belonging to the genus *Sporothrix*, commonly seen in tropical and subtropical regions like Madagascar. This study describes the epidemiological, clinical, and the treatment outcomes with itraconazole for sporotrichosis.

**Methods:**

A descriptive retrospective study on patients suffering from sporotrichosis, from March 2013 to January 2019, was conducted, in the reference center for endemic mycoses. Patients with sporotrichosis who received itraconazole for at least 3 months were included in the study. Patients received itraconazole 200 mg daily for 3 to 6 months. Therapeutic responses were evaluated at months 3 and 6 after treatment. Outcomes were classified as minor response, major response, cure, and failure.

**Results:**

Forty-three cases of sporotrichosis were included. The median age of patients was 40 years. Most of them (62.8%) were rural workers. Men were more frequently infected (72%). At the end of 6^th^ month of treatment, 20 patients (46.5%) were cured and a major response to itraconazole was observed in 8 patients (18.6%). The cure rate was higher in 12 patients (27.9%) who had disease durations of less than to one year than in those who had more time of disease (18.6%) (p = 0.01). Thirteen patients (30.2%) were lost to follow up. The rate of lost to follow-up was also higher (39.5%) in patients who had sporotrichosis of less than to one year than in those who had more time of disease.

**Conclusion:**

The cure rate found in this study was inferior to that reported in the literature. However, it was higher in patients with early symptomatology.

## Author summary

Sporotrichosis is a subcutaneous mycosis, more common in tropical and subtropical countries. It is endemic in Madagascar and occurs following injury and telluric contamination. We evaluated the therapeutic response to itraconazole in 43 cases of sporotrichosis seen at the reference center in dermatology, Madagascar. At the end of 6^th^ month of itraconazole therapy, the cure rate was 46.5%. Major response to itraconazole was observed in 18.6% of patients. Patients with sporotrichosis onset in less than a year had a high cure rate (27.9%) compared to other patients (18.6%). In this study, high rate of lost to follow-up was found, correlated to the disease duration. Our study highlighted the difficulty encountered during its management.

## Introduction

Sporotrichosis (SPT) is a subacute to chronic granulomatous mycotic infection usually involving the skin and subcutaneous tissues. It is an implantation mycosis (fungal disease that develop at the site of transcutaneous trauma), caused by dimorphic fungi belonging to the genus, *Sporothrix*, which are distributed over tropical and subtropical regions of the world. SPT is primarily an occupational disease that may infect agricultural or forestry workers and others involved in outdoor activities after transcutaneous inoculation. SPT mainly affects populations living in remote areas, where diagnosis and treatment is frequently delayed [1,2]. SPT can affect also several species of mammalian animals. Transmission mainly from cats and feline can carry large amounts of yeast to their close contact with humans, allowed the characterization of SPT as a zoonosis [3]. Brazil is one of the areas who has experienced geographic expansion of zoonotic sporotrichosis in the last decades [4].

Spontaneous regression or remission is uncommonly seen and treatment is required for most patients. The management of SPT requires long-term administration (3–6 months) of antifungal therapy. Since the 1990s, azoles have been used. Currently, itraconazole remains the drug of choice for the treatment of sporotrichosis [5].

In Madagascar, most of the SPT cases were concentrated in the highlands, with an average annual prevalence of 0.21 cases/100,000 inhabitants. It may be explained by the climate conditions in this region characterized by a mean temperature of 19.5°C and substantial rainfall, which probably favors development of fungi. Young persons (<18 years of age) had high infection risk [6]. The purpose of this study is to offer an overview of SPT in Madagascar based on a case series. It focuses mainly on the clinical and therapeutic response of this disease.

## Methodology

### Ethics statement

This study was performed in accordance with the Ethics Committee for Biomedical Research of the Ministry of Public Health of Madagascar (authorization no.66-MSANP/CE). Written informed consent was obtained from all participants and parents for child participants.

A retrospective study was conducted in the department of Dermatology at the University hospital of Antananarivo, Madagascar and during consultation campaigns in districts (in the central highlands, the north, south, and north-west of Madagascar). Fifteen patients were found as part of the previous study conducted by Rasamoelina et al, and that records were checked retrospectively.

Patients with clinically suspected sporotrichosis seen between March 2013 and January 2019 were recruited. Patients who presented with confirmed, probable and possible SPT and who had received itraconazole for at least 3 months were included in this case series. Confirmed, probable and possible SPT cases were defined as previously described [6], summarized in Table 1.

**Table 1:** Clinical, mycological and histological classification of SPT.

Criteria	Major	Minor
**Clinical criteria**	Lymphocutaneous SPT defined as a papule or pustule or a subutaneous nodule at the inoculation site, then ulceration with erythematosus edges and purulent secretion. Secondary lesions arise along the path of regional lymphatic vessels	Mucosal SPT: nasal septum, with bloody secretions and detachment of crusts. Conjunctivitis with granulomatous lesions accompanied by a serous-purulent discharge
	Fixed or cutaneously disseminated	Primary pulmonary SPT. Radiological patterns include cavitary disease, trecheobronchial lymph node enlargement, and nodular lesions.
	Extracutaneous SPT: disseminated, osteoaticular, ocular	Vegetative or verruccous lesions, infiltrated plaques, tuberous lesions
**Histological and mycological criteria**	Identification of *S*. *schenckii* by PCR with specific primers or ITS sequencing, directly from clinical samples or from a positive culture of a fungus morphologically suggestive of *Sporothrix spp*	« Cigar-shaped yeast » observed on direct microscopic examination or histologic analysis
	MALDI TOF mass spectrometry identification of *S*.*schenkii* from a positive culture of a fungus morphologically suggestive of *Sporothrix spp*	« Asteroid bodies » in direct examination of pus and/or histology analysis (Splendore-Hoeppli reaction)
		Positive culture of a fungus morphologically suggestive of *Sporothrix spp* from a clinical sample without molecular or MALDI TOF mass spectrometry confirmation
**Classification of cases**		
**Confirmed**	≥1 of the major clinical criteria and ≥1 of the major mycologic criteria or 1 minor clinical criterion and ≥1 of the major mycologic criteria	
**Probable**	≥1 of the major clinical criteria and 1 minor mycologic or histologic criterion and a complete or partial response to antifungal therapy	
**Possible**	≥1 of the major clinical criteria without any (major or minor) mycologic or histologic criteria or ≥1 of the minor clinical criteria without any (major or minor) mycologic or histologic criteria and a complete or partial response to antifungal therapy	

The following baseline characteristics were recorded:

- Demographic aspects: age, gender, occupation
- Epidemiological aspects: probable area of contamination, type of trauma
- Clinical aspectcs: anatomical site, severity of lesions, disease duration (months), clinical classsification (fixed cutaneous or lymphocutaneous)
- Therapeutic aspects: doses and time of treatment (months), adverse events
- Outcomes

Mycological analysis and molecular analysis were performed to identify the aetiological agent. For mycological analysis, microscopic examination was carried out for all clinical specimens collected, with or without Chlorazol Black staining. Isolates were stained with lactophenol blue. Samples were then seeded in test tubes with Sabouraud medium supplemented with chloramphenicol and incubated at 30°C for two to three weeks. The growth of the cultures was supervised during these incubation times. If culture was positive, the morphological aspect was analyzed. Macroscopic and microscopic examination of cultures allows just to suspect *Sporothrix sp*. Structures do not permit the confirmation of *S*. *schenkii*.

For molecular analyses, QIAamp DNA Blood Mini Kit (https://www.qiagen.com) were used according to the manufacturer’s instructions for DNA extractions. Colonies and biopsies were crushed before processing. Panfungal PCRs targeting internal transcribed spacer (ITS) regions with the primers ITS1/ITS4 and D1D2 with the primers NL-1/NL-4 and NL-3/NL-4 were performed in the first step [7, 8]. This was followed by a specific *S*. *schenckii* PCR targeting the topoisomerase II gene with SSHF31/SSHR97 primers in the second step. Panfungal PCR products were sequenced via LGC Genomics GmbH by using ITS1/ITS4 primers [9].

### The therapeutic response assessment

Patients received itraconazole 100mg twice a day at least for 3 months, as part of their care.

Clinical response to itraconazole was assessed in the 3^rd^ and 6^th^ months. It was classified into four groups:

- cure: lymphangitis and subcutaneous nodule remission; absence of erythema and infiltrates; total epithelialization
- major response: partial healing, lesions reduced in size by more than 50% of their initial size
- minor response: mild improvement of most lesions, a smaller decrease in subcutaneous nodules than for a major response
- failure: absence of therapeutic response.

Clinical assessments were performed by dermatologists every month after the initiation of treatment. Clinically, the adverse effects of treatment were assessed. A follow-up of 6 months after starting itraconazole was planned in order to evaluate the rate of relapses.

Informed consent was obtained from all participants. Patients’ data was kept anonymous to ensure confidentiality and privacy.

### Statistical analysis

For data processing and analysis, Epi info version 3.5.4 software was used. The chi-square (X^2^) and Fischer exact were used to analyse qualitative variables. Significance levels p lower than 0,05 were considered for the analyses.

In the multivariate analysis, all epidemiological and clinical parameters were added as covariates and compared with one another. The odds ratio (OR), 95% confidence intervals (CI) and p values were used as estimates of the effect of each variable.

## Results

Forty three patients with SPT were included in this study. The mean age of patients was 40.09 years (range: 10–78 years). Male predominance was observed with a sex ratio M/F of 2.5.

The farmers’ and forest technical officers’ represented 62.7% of cases. Thirty four (74.4%) patients were living in the Madagascar highlands, corresponding to the presumed area of contamination. Seven patients mentioned trauma by telluric agents. Other patients don’t remember or have neglected trauma prior the apparition of SPT lesions.

There are 35 confirmed SPT cases, 2 probable SPT cases and 6 possible SPT cases.

The lesions were more localized on the upper limbs in 23 (51%) patients and on the lower limbs in 18 (41%) cases. The clinical lymphocutaneous form was the most frequent (38 cases). Four patients presented fixed forms and one patient presented disseminated forms. Sixty-nine percent of patients had a time from onset to diagnosis of less than one year. The distribution of participants by sociodemographic and clinical characteristics is shown in Table 2.

**Table 2:** Distribution of participants by sociodemographic and clinical characteristics.

Characteristics	n(%)
**Age, years**	
10–29	5 (11.6)
30–49	15 (34.8)
50–69	16 (37.2)
>70	7 (16.2)
**Gender**	
Male	31 (72)
Female	12 (27.9)
**Occupation**	
Farmer	27 (62.7)
Services sector	13 (30.2)
Student	3 (6.9)
**Anatomic location**	
Lower limb	18 (41.8)
Upper limb	23 (53.4)
Cephalic	1 (2.3)
**Duration of the lesions, years**	
<1	30 (69.7)
1–2	6 (13.9)
>2	7 (16.2)
**Clinical form**	
Lymphocutaneous	38 (88.3)
Cutaneous	4 (9.3)
Disseminated	1 (2.3)

Macroscopic and microscopic examination of cultures of all patients’samples exhibited morphologic features consistent with *Sporothrix spp*.

*Sporothrix schenckii* was the only fungal species isolated in 35 strains by MALDI-TOF mass spectrometry, in this study. Among these, 30 were also identified by the specific S.schenkii topoisomerase II PCR and 10 were also identified by ITS sequencing.

### Therapeutic responses

**Fig 1:**
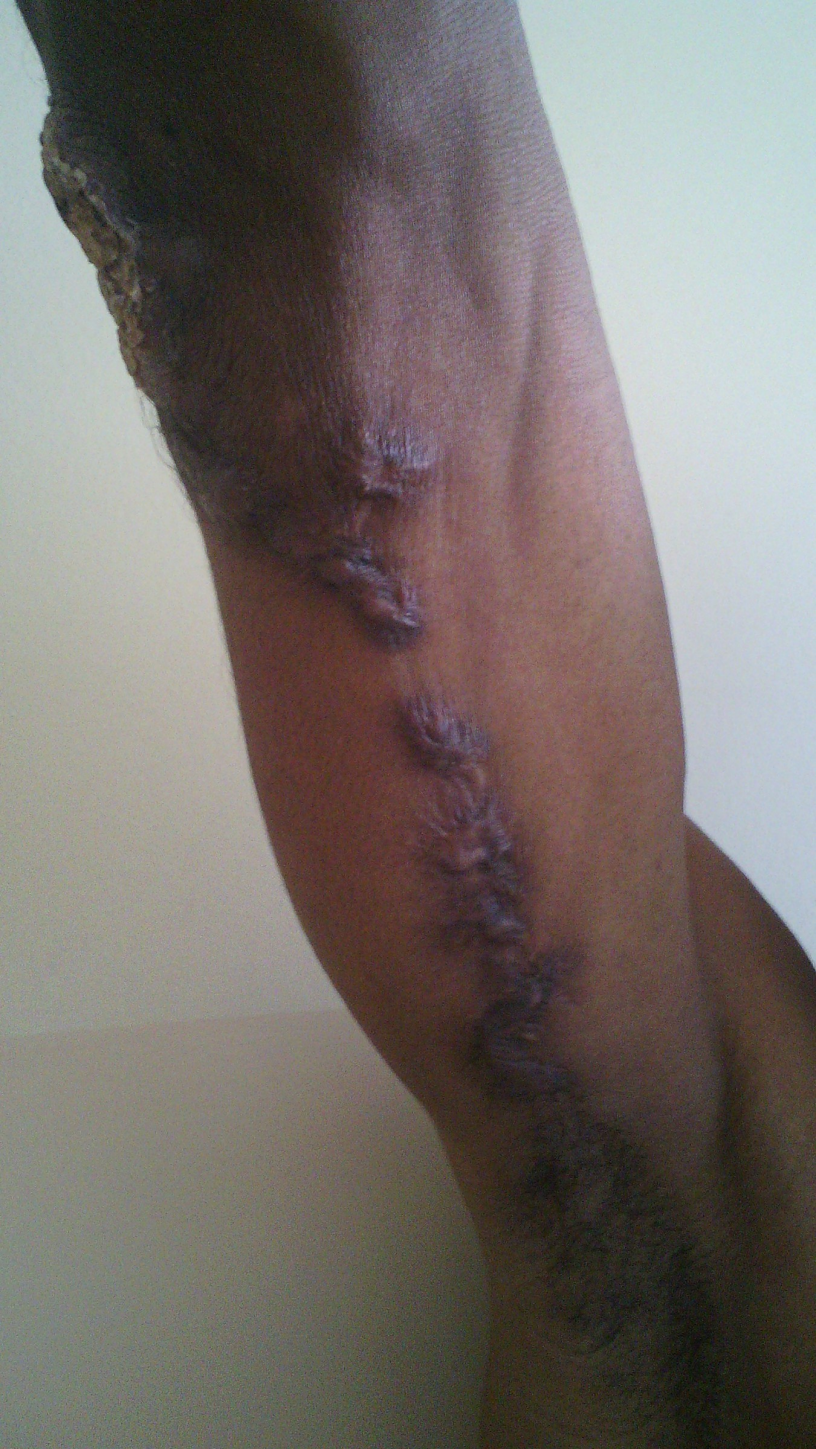
Lymphocutaneous SPT on the left upper limb before starting treatment.

**Fig 2:**
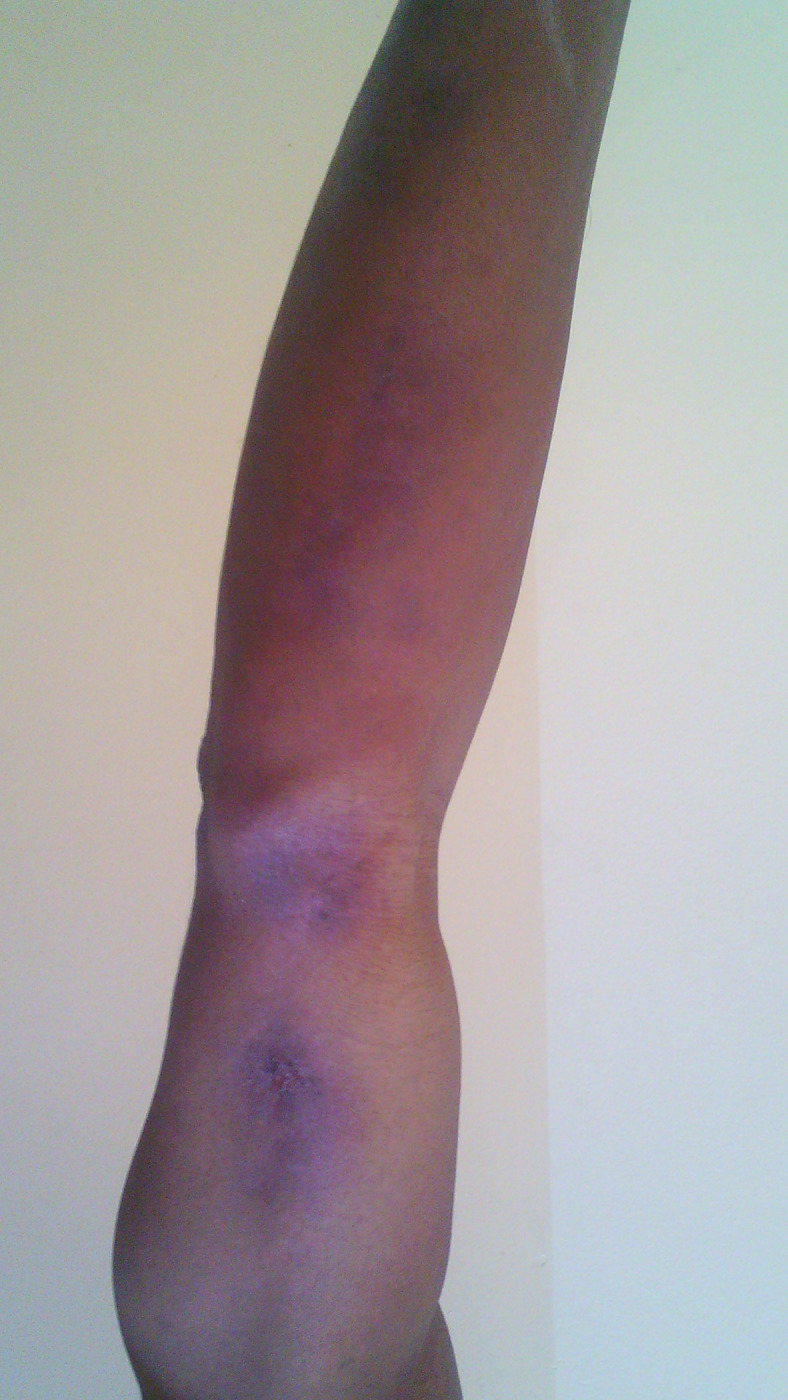
Major response of lymphocutaneous SPT on the left upper limb at the end of 6th month of itraconazole.

**Fig 3:**
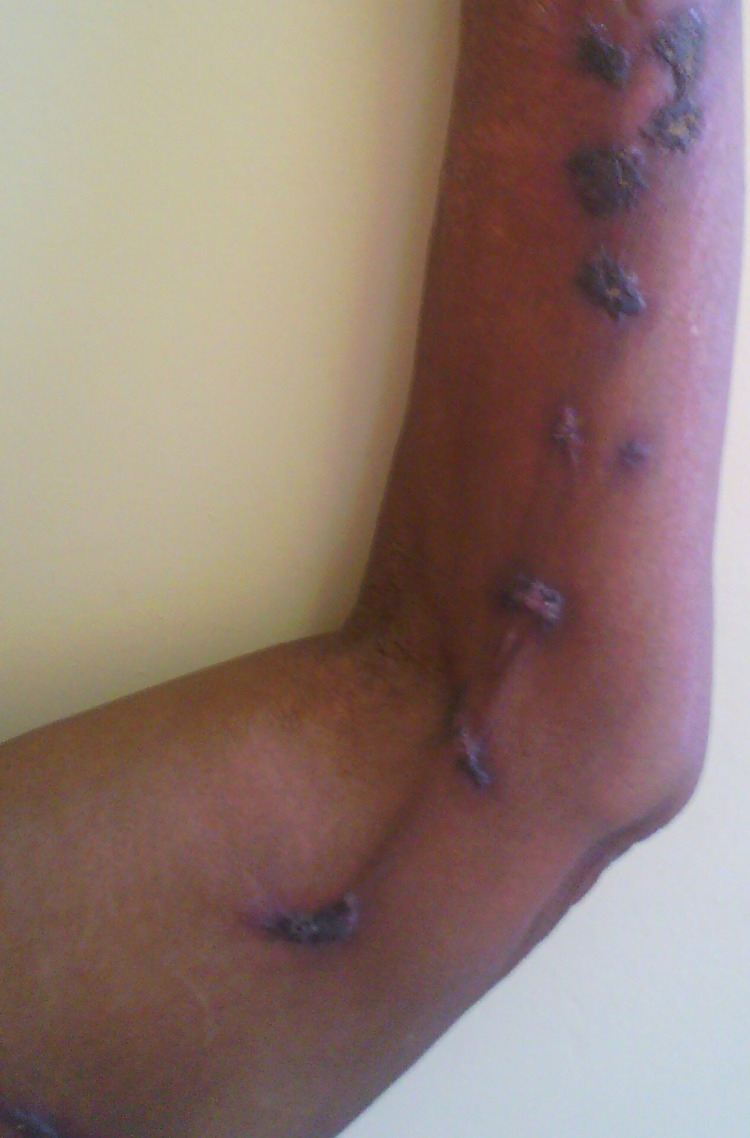
Lymphocutaneous SPT on the left upper limb before starting treatment.

**Fig 4:**
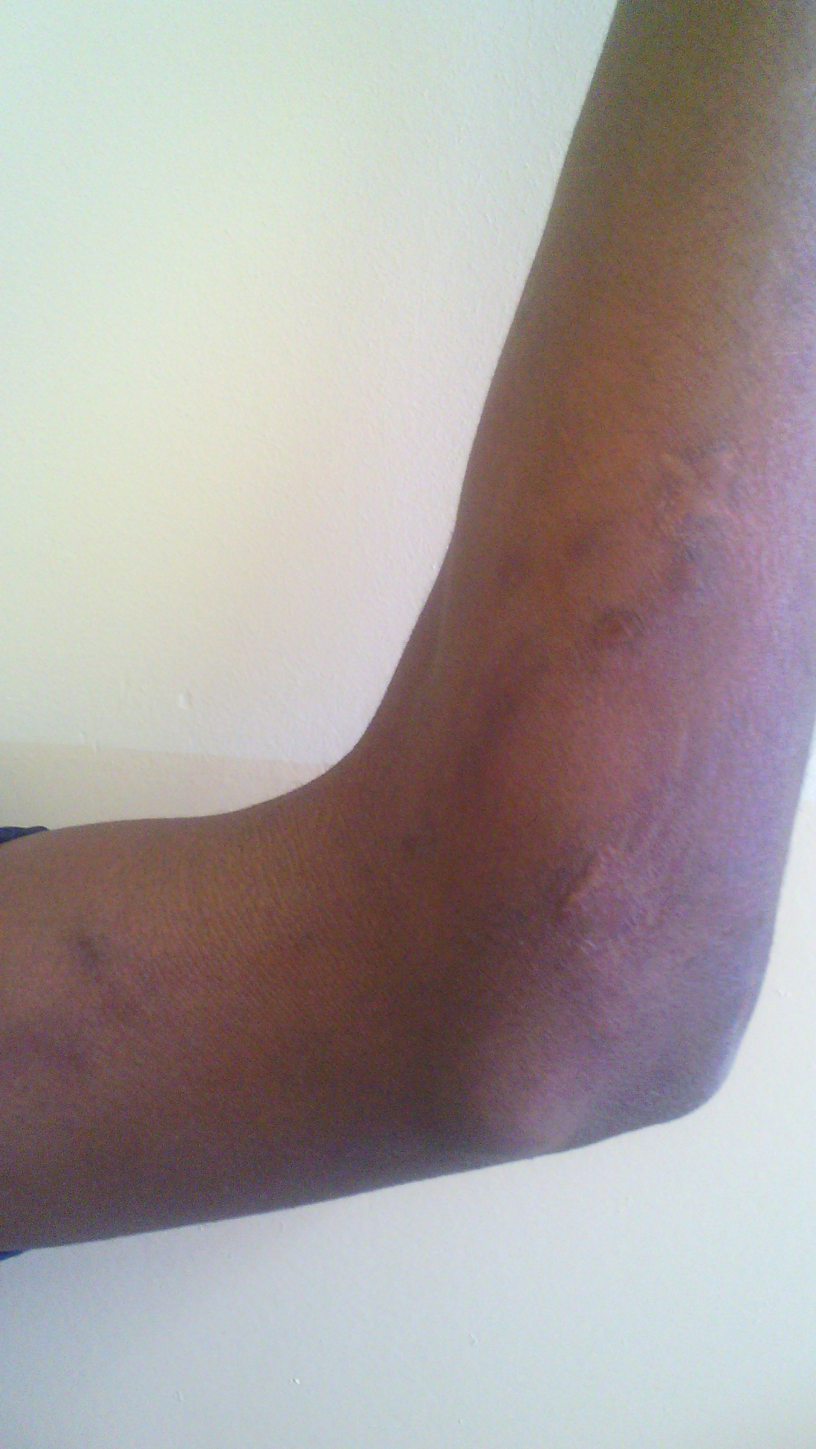
Cure of lymphocutaneous SPT on the left upper limb at the end of 6th month of itraconazole.

No patient was cured before the end of the 3^rd^ month. Therapeutic assessment at the end of the 3^rd^ month showed minor response in 34.9% of cases, major response in 62.8% and cure in 2.3% of cases. The repartition of therapeutic responses at the end of the 3^th^ month is shown in Table 3. Cure rate increased at the end of the 6^th^ month at 46.5%. Thirteen patients were lost of follow-up after 3 months of treatment. The repartition of therapeutic response at the end of the 6^th^ month is shown in Table 4. Figs 1 and 2 show the major response to itraconazole for lymphocutaneous sporotrichosis on the upper limb after 6 months, while Figs 3 and 4 show total recovery of lesions in another patient. Neither adverse effect, nor a failure to itraconazole was found.

**Table 3:** Therapeutic response at the end of the 3^rd^ month.

Parameters	Minor response	Major response	Cure	p-value
**Age**				
[10–40]	9	12	0	0.19
[40–70]	6	13	1	
≥ 70	0	2	0	
**Gender**				
Male	12	18	1	0.17
Female	3	9	0	
**Occupation**				
Farmers	7	19	1	0.05
Others	8	8	0	
**Previous antifungal therapy**				
Yes	0	2	0	1
No	15	25	1	
**Duration of disease (years)**				
<1	11	18	1	0.03
1–2	2	4	0	
>2	2	5	0	
**Topography**				
Cephalic	1	0	0	0.03
Upper limb	7	16	1	
Lower limb	7	11	0	

**Table 4:** Therapeutic response at the end of the 6^th^ month.

Parameters	Minor response	Major response	Cure	p-value
**Age**				
[10–40]	1	4	10	0.99
[40–70]	1	4	9	
≥ 70	0	2	1	
**Gender**				
Male	1	7	13	0.1
Female	1	1	7	
**Occupation**				
Farmers	1	4	13	0.5
Others	1	5	7	
**Previous antifungal therapy**				
Yes	0	1	0	0.18
No	20	8	20	
**Duration of disease (years)**				
<1	1	8	12	0.01
1–2	0	0	3	
>2	1	1	5	
**Topography**				
Cephalic	0	1	0	0.04
Upper limb	1	4	1	
Lower limb	1	4	0	

### Factors affecting therapeutic response at the end of the 6^th^ month

No correlation was found between age, gender, profession and therapeutic response (p = 0.99, p = 0.1 and p = 0.5, respectively).

Good therapeutic response was seen in SPT localized in the upper limb than in lower limb and cephalic lesions (p = 0.04).

The cure rate was higher in patients who had a disease duration of less than one year than in those who had more (p = 0.01).

No correlation was found between prior antifungal therapy and therapeutic response.

In the multivariate analysis, our result revealed that therapeutic response was linked to the disease duration (OR 6.7; IC 95% [0.67; 0.99]; p = 0.04) (Table 5).

**Table 5:** Summary of multivariable regression analysis.

Covariate	OR	CI	p value
**Minor/Major response vs Cure**			
**Age group**			
[10–40]	1	[0.16; 5.9]	1
[40–70]	1.21	[0.2; 7.25]	1
>70	2.05	[0.02; 174]	1
**Male**	2.1	[0.29; 25.7]	0.67
**Farmers**	0.46	[0.07;2.56]	0.44
**Duration disease < 1 year**	6.7	[0.67; 0.99]	0.04
**Topography in lower limb**	0.69	[0.007; 65.2]	1

### Predictors of lost to follow-up

Thirteen patients were lost to follow-up after 3 months of treatment. Age and gender had no effect on losing to follow-up. The disease duration, on the other hand, had an effect: the rate of lost to follow-up was higher in patients who presented SPT less than one year than in the other group (OR 30.14; IC 95% [3.29–15.2]; p< 0.001). Table 6 shows the predictors of loss to follow-up.

**Table 6:** Predictors of loss to follow-up.

Parameters	Lost to follow-up in the 6th month		p value
	Yes	No	
**Gender**			
Female	7	9	1
Male	16	22	
**Occupation**			
Farmers	15	18	0.5
Others	8	5	
**Duration disease**			
<1	17	21	0.002
1–2	4	3	
>2	2	7	

## Discussion

The present study reported the therapeutic response of 43 cases of SPT to itraconazole, seen in Madagascar. Even though there were good clinical outcomes, the high rate of loss to follow-up was a limitation of the study.

Our case series revealed considerable involvement in rural activities. Not only farmers and forest technical officers are exposed to contamination; people in the services sector are also exposed because they practice agriculture in partial time for living.

Itraconazole was administered at a dose of 100–200 mg daily, orally for at least 12 weeks in SPT [10]. Several authors recommend starting sporotrichosis treatment with 200mg/day [10, 11, 12]. Other authors has proved that the regimen with 100 mg/day was effective and safe, in addition to having a lower cost [13]. Itraconazole is efficacious and well-tolerated, which has essentially replaced amphotericin B and supersaturated potassium iodide (SSKI) in cutaneous and extracutaneous sporotrichosis due to its 90–100% efficacy rates [14]. In our study, itraconazole was used at a dose of 200 mg daily, with a mean duration of treatment of 6 months. The cure rate was 2.3% at the end of the 3^rd^ month, 46.5% at the end of the 6^th^ month. Our result was inferior to that reported by other studies. Sharkey-Mathis et al. reported 27 cases of SPT treated by itraconazole of 100 to 600 mg daily for 3 to 18 months; 25 cases had a good response to itraconazole and failure was observed in 5 cases [15]. A large study with 645 cases of SPT found an excellent therapeutic response with the minimum dose of itraconazole (50 to 400 mg daily) and there was a low incidence of treatment failure; only 26 patients were lost to follow-up and 9 cases were switched to other antifungal agents and substituted for thermotherapy [13].

This difference may be explained by the high rate of patients lost to follow-up in our case series. In fact, a third of our patients didn’t have a clinical evaluation at the end of the 6^th^ month.

### Factors related to therapeutic response

As well as in previous studies, this present study shows no correlation between age, gender, profession and therapeutic response to itraconazole in SPT. However, the duration of disease appeared to be significantly related to the therapeutic response. The cure rate was higher in patients with symptomatology for less than a year than in another group (p = 0.04 according to multivariate analysis). This relationship could be explained by the fact that a recent infection is less extensive and not complicated. So, correct diagnosis and early management of SPT are therefore essential. Other factors such as therapeutic adherence influenced by social and economic problems, and accessibility to health care may influence also this finding.

### Factors related to loss to follow-up

After 3 months of treatment, 30% of cases were lost to follow-up. The rate of lost to follow-up in this study is higher than that reported in Brazil (21%) [16] but lower than in Peru (34.1%) [17]. Our study found that the duration of symptomatology of more than one year was a significant risk factor for being lost to follow-up (p = 0.0002; OR 30.14; IC 95% [3.29–15.2]). Geographical remoteness and lack of financial resources are the main barriers to accessing health care in Madagascar. Furthermore, clinical improvement was seen in 62,8% of cases at the end of the 3^rd^ month. This result may have an impact on the follow-up rate at the 6^th^ month because partial healing could mislead patients to stop treatment.

## Conclusion

Sporotrichosis remains a public health problem and a neglected disease in Madagascar. Not only may sporotrichosis generate personal disabilities, but also its management needs social and financial resources. In this study, the cure rate was 46.5% at the end of the 6^th^ month. The cure rate was higher in patients with early symptomatology. Management’s strategies should be improved in order to achieve more therapeutic results. Our study highlighted the difficulty encountered during its management, particularly during the follow-up. Not only were patients seen at an advanced stage, but they had difficulty maintaining long-term treatment. The lack of technical platforms in endemic areas and the high rate of loss to follow-up determined the therapeutic difficulty of SPT in Madagascar.
